# A qualitative study on existential suffering and assisted suicide in Switzerland

**DOI:** 10.1186/s12910-019-0367-9

**Published:** 2019-05-14

**Authors:** Marie-Estelle Gaignard, Samia Hurst

**Affiliations:** 1 Institute for Ethics, History, and the Humanities, Institute for Biomedical Ethics, Geneva University Medical School, Rue Michel-Servet, 1, 1211 Geneva, Switzerland; 2 Department of Oncology, Geneva University Hospitals, Rue Gabrielle-Perret-Gentil 4, 1205 Geneva, Switzerland

## Abstract

**Background:**

In Switzerland, people can be granted access to assisted suicide (AS) on condition that the person whose wish is to die performs the fatal act, that he has his decisional capacity and that the assisting person’s conduct is not selfishly motivated. No restrictions relating to the ground of suffering are mentioned in the act. Existential suffering as a reason for wanting to die, however, gives raise to controversial issues. Moreover, existential suffering lacks definition and no consensus exists on how to evaluate and manage it. This study explores the perspectives of care professionals and volunteers from a “right-to-die organization” on existential suffering as a motive for assisted suicide requests.

**Methods:**

A qualitative study based on face-to-face interviews was conducted with twenty-six participants: palliative care and primary care providers as well as EXIT right-to-die organization volunteers. Elements from the grounded theory approach were used.

**Results:**

The twenty-six participants described existential suffering in a multiplicity of individual ways. In total, sixty-three stories were recounted. Their representations were grouped into eight categories: physical decline and its consequences, loneliness, fear of the future, life is over, loss of social significance, loss of hope for a better future, being a financial burden and loss of pleasurable activities. According to all participants, suffering coming from the loss of self-identity was always linked to physical decline, as if one’s image completely defined someone’s identity. Society’s perception of old people and vulnerable people were also often questioned. Another interesting point was that only four stories referring to a “pure” existential suffering were found. This suffering was related to a feeling that life has come to an end, without identification of any other related restriction or suffering.

**Conclusions:**

Existential suffering is multifaceted. Legislators and right-to-die organisations have to address the question of what make a AS acceptable. The plurality of existential suffering implies the need of a very personalized care. A better understanding of what it is made of could provide a “toolbox” to people concerned by these requests, helping them to explore it in order to offer suffering people a wider range of alternatives.

**Electronic supplementary material:**

The online version of this article (10.1186/s12910-019-0367-9) contains supplementary material, which is available to authorized users.

## Background

Existential suffering is frequent in seriously ill patients [1], an important factor affecting quality of life [2], one of the reasons why some patients request assisted suicide (AS). [3] The acceptability of requests for AS motivated by existential suffering are, however, controversial. [3–6] This controversy is rendered more difficult by vagueness regarding what people understand the definition of existential suffering to be. Indeed, it is often simply described as “being tired of life”, “distress” that arises when the meaning and value of one’s life is unclear » [7], and despite the increasing focus on existential concerns over the past decade, its definition still lacks consistency and there is no consistency on how to evaluate or treat it. A recent article reviewed the definition of existential suffering and suggested that it could be understood as an incapacitating state of despair resulting from an inner realization that life is futile and without meaning. [8] In 2011, a review identified 56 definitions of the term “existential suffering”. [9] The authors emphasized relevant themes such as: the loss of meaning or purpose in life, hopelessness, feeling of loneliness, fear of being a burden, fear of future, loss of social role functioning. They also pointed out the difficulty in distinguishing existential suffering from spiritual concerns or from psychological symptoms such as depression.

Switzerland has what may well be the most liberal legislation on AS in the world, making the question of where to set the limits in practice even more relevant. [10] Only three conditions are needed in this country to access AS: first, the person whose wish is to die has to perform the fatal act; second, this person has to have her/his decisional capacity; finally, the conduct of the assisting person must have no selfish motive. It should also be noted that the prescription of lethal drugs are subject to the Federal Narcotics Act. [11] This law leaves an important place to individual freedom and permits the “right-to-die organizations” in Switzerland to define the boundaries of their practice within the legal framework. In 2016, 928 people died from AS in Switzerland and a 2013 study showed that physician-assisted death counted for 1.1% of all death. [12, 13] Although the legislation does not examine the reason for requesting AS, the motive “existential suffering” often gives raise to controversial issues. What, then, makes a case of AS morally acceptable? Suffering is surely not limited to disease status. If we consider relief from suffering to be one of the central considerations for AS, it is reasonable to think that the acceptability of a request should not exclusively depend on the diagnosis of an incurable or terminal disease. [14, 15]

Professional definitions of existential suffering were examined in 2004 [16], but to our best knowledge no study has been performed about their perspectives when this existential pain or suffering is part of the request for AS in a country where it is legal. Moreover, no study has been conducted to see how those people confront it, how they actually manage this kind of suffering. This is why, in the context of this particular legislation, we asked care professionals and volunteers from a “right-to-die organization” (EXIT Suisse Romande) who are confronted to assisted suicide to tell us about their experiences regarding existential suffering as being the reason for the request. Asking them first about their representations of existential suffering then enabled us to progressively build the conversation around their attitudes when confronted to it. We hope that their responses can be used to inform the debates in Switzerland and elsewhere about assisted suicide and in particular about the controversy around the acceptability of existential suffering as being the reason for an AS request. In this paper, we present the first part of our study. Before discussing the acceptability of existential suffering as a motive for AS requests, it is essential to clearly understand what this kind of suffering is and who are the people who endure it and request AS. This study could also stimulate thoughts about preferable alternatives that could be offered to these suffering people.

## Methods

### Study design

We chose a qualitative design, face-to-face interviews, for this study because of the lack of insights on how to manage existential suffering when it’s part of the request for assisted suicide. Elements from the grounded theory approach were used to develop a conceptual model derived from the data.

### Sample

We contacted a full spectrum of persons that we thought to be the most commonly involved in end-of-life care and assisted suicide and we included them into three groups (volunteers from a Swiss “right-to-die organization”, palliative care providers and primary care providers). A snowball sampling was used and each participant was contacted either by email or phone and explanations were provided regarding the goals and the modalities of our study. We also asked them if they felt concerned by this issue and if they consented to participate. No additional selection criteria was taken into account.

### Data collection

After this first contact, we collected data through face-to-face interviews done by the main research at a location chosen by the participant (most of the time at their office). Concise information about the study was given before an informed consent was signed (written consent). At the beginning of each interview, the participants were asked their age, gender, job, and how many years of experience they had. We used a semi-structured interview guide (Additional file 1: Table 1). Interviews ranged from twenty to fifty minutes depending on participants’ experiences with deliberating about assisted suicide and existential suffering. All interviews were tape-recorded and then transcribed verbatim.

### Data analysis

We proceeded in three steps: open coding, axial coding and selective coding. As a first step, we used open coding which means breaking down data to examine and conceptualize it into codes. We therefore labelled phrases from the participants. In this example of open coding, a palliative care provider talks about a patient she used to see at home because of chronic pain and who one day shared with her her desire to request AS: “… and who had planned for a long long time to call EXIT with the explanation that she finally had very little company, that her existence didn’t serve anybody anymore and that it finally was her only option.” We labelled this phrase as an example of “loneliness”, “feeling useless” and “loss of hope for a better future”. Three codes were used here.

Axial coding was the second step of our analysis. It consisted of reassembling our codes into broader categories in order to better understand the facets and associations between them. We for example associated the codes “physical decline”, “dependency” and “hurt self” found in different testimonies as being part of the wider category “physical decline and its consequences”. Indeed, according to all participants dependency and hurt self were always a consequence of physical decline.

Selective coding was used only for the code “life is over” because it was found to be a core category and it was important to better clarify its relationship with the other codes.

Two full recordings were double coded and discussed together at different stages during the analysis, to ensure that codes well represented the testimonies of the participants and were suitably associated with others into wider categories. After coding 21 interviews, no newer codes appeared and thus we were satisfied that data saturation had been reached for the main results presented here. [17, 18] All quotations were translated from the original French by the authors. People identification is mentioned as follows: Pn (Participant number) / PallCP (Palliative Care Providers) or PrimCP (Primary Care Providers) or EV (EXIT Volunteer).

### Protection of human participants

This study was exempted from ethics review by the president of the *CCER* (Commission Cantonale d’Ethique de la Recherche, Swiss Cantonal Ethics Committee) because the study entailed no more than minimal risks and was outside the scope of the Swiss Federal Act (Human Research Act, HRA) on research with human participants.

The participation was voluntary and did not involve the collection of personally identifiable information.

## Results

### Participants

Participant characteristics are described in Additional file 1: Table 2. Between January and April 2016, twenty-seven professionals were contacted to participate in this study. Only one did not answer our request, therefore twenty-six people participated altogether. This included four physicians, one nurse, one physiotherapist, one nurse’s aid, two psychologists, one social worker and two hospital chaplains of a palliative care unit. In addition, we interviewed six volunteers from a Swiss “right-to-die organization” (*EXIT Suisse Romande*), one physician and three nurses from a palliative care mobile team, two general practitioners and a nurse, as well as the person responsible for recreation in a retirement home. The participants varied in gender, age and years of experience.

### Professionals’ perspectives on existential suffering as a reason for requesting assisted suicide

Providers from palliative care and primary care, as well as volunteers from *EXIT*, described existential suffering in a multiplicity of very individual ways. In total, they recounted sixty-three stories. When referring to existential suffering, most of them described a life that wasn’t worth living any longer and/or a life that did not make sense anymore, for many different reasons. The coding of their statements resulted in eight categories that were ranked according to the frequency of their occurrence: physical decline and its consequences, loneliness, fear of the future, life is over, loss of social significance, loss of hope for a better future, being a financial burden, and loss of pleasurable activities (Additional file 1: Table 3).

### Physical decline and its consequences

A vast majority of the participants referred to experiences regarding physical decline and feelings that can result from this decline. The codes grouped in this category are thus: physical decline itself, dependency and hurt self. Each of those includes sub-codes that are listed in Additional file 1: Table 3.

Physical decline itself and dependency were the two most represented codes in this category. Below is an example of how physical decline can lead to a request for AS:

*“( …*) *but finally the problem (for her) was the anal incontinence, she didn’t want to continue (living) so she asked us (to assist her) and told us: “Now it’s my limit. I am already blind, deaf, I can’t move anymore and now this (the incontinence). That’s enough!”” (P19/EV).*

According to this participant, the reason why this person wanted to die was because of a too damaged physical condition. Actually, most of the participants considered physical decline as being a trigger for existential suffering, in that it induces dependency, pain, loss of pleasurable activities, hurt self or loss of hope. Moreover, we found that the idea of dependency was always linked to physical decline and this is why dependency was included in this category. A palliative care provider illustrates it:

*“On the other hand, I had the feeling that for this woman, beyond a certain limit, it was no longer acceptable. She was too damaged, too dependent. You could see it. So, she preferred stepping down before becoming too dependent on nursing.” (P15/PallCP).*

This quote shows that dependency was actually considered by this participant as unbearable. It was relevant in many other testimonies.

Participants also pointed out the fact that dependency can induce a diminished perception of someone’s own image and that suffering originates from this feeling. “Hurt self”, one of the codes in this category, is illustrated in the following example:

*“The caregivers also play an important role because they frequently are the confidants of patients during the bathing, during their physical activities. It’s often here that patients talk about their suffering regarding their loss of autonomy, their physical decline. It’s often what we hear. One’s self-image also plays an important role in this kind of decision. Patients often tell us: « I don’t recognize myself ».” (P16/PallCP).*

“I don’t recognize myself” puts into question the notion of self-identity and it was actually always mentioned this way. This statement signal the role of one’s image or physical appearance defining one’s identity. All participants spoke of deteriorating physical appearance as inducing a loss of one’s self identity. Another participant expresses it differently while talking about an illness that can be the turning point of a change in self-identity:

*“There is a relevant element. It’s that often the illness, the severe illness, irrupted into these people’s life and this constituted a* threshold*. And rightly there often is a fracture between the “before of who I was once” and the “now” where people often take an « ill person » identity. And their identity is now limited to the issue of being ill.” (P8/PallCP).*

When participants talked about physical decline, dependency or hurt self, there was another relevant idea to point out. The fact that all the sufferings induced by those conditions can be partly explained by how people perceive them. Here is an illustration by one palliative care provider:

*“( …*) *I’m convinced that the way society perceives those people who are not productive anymore, ( …*), *with cognitive trouble, (those people) becoming a financial burden and the ones becoming diminished or incontinent, is a problem.” (P10/PallCP).*

This notion that how people perceive old age or dependency is to some extent responsible for those multiple and different types existential sufferings was recurrent.

### Loneliness

Loneliness was the second most mentioned component of existential suffering with nearly two-third of participants who talked about it. According to many participants, loneliness seems to be a significant cause for requesting AS. This is illustrated by a general practitioner talking about one of the last discussions she had with one of her patients:

*“( …*) *and I told her "I’d just like to know something honestly. If you were with children, grandchildren, would you do that? (request AS). She told me “Surely not”; and I told her “So, it is really (because of) loneliness?” And she told me “Yes”” (P24/PrimCP).*

Loneliness was also often described as a loss of human contact. A hospital chaplain talked about solutions that could be proposed to people demanding AS due to existential suffering in these terms:

*“So, I think of the possibilities, of activating a greater human proximity, because it’s actually often what is lacking. This lack of connection, this lack of contact. But it is also because this is what people often wanted. Because they arrived to this type of loneliness.” (P5/PallCP).*

She actually referred to a kind of “chosen loneliness” contrary to the one induced by the loss of a loved one. Another participant illustrates it:

*“She didn’t necessarily have pain, but it’s true that her treatment was quite heavy for her. And in addition, the fact that she was deeply mourning her husband. So, it’s true, I don’t know what exactly motivated her wish to commit suicide”. (P6/PallCP).*

This palliative care provider did not exactly understand why his patient requested AS but suggested that loneliness might be partly responsible for it.

### Fear of the future

The codes of this category are: fear of a terrible agony, fear of being placed into a retirement home, fear of the unknown, fear of the hospital and anxiety. The fear of an imagined agony was linked most of the times to past experiences, as a nurse put it:

*“By digging a bit what was worrying him, it was the memory of his spouse who died from an oncologic illness. She was very young at the time and she was screaming out in pain. And he was leaving the house in order to not hear her screaming out of pain. This had a big impact on him. His fear was really linked to the fact that medicine couldn’t address many of the symptoms, pain in particular.” (P11/PallCP).*

The fear of being placed into a retirement home was also expressed a few times and was often linked to the fear of physical decline and dependency as a general practitioner explained:

*“( …*) *and they had talked about a placement in a retirement home. And I’d say that for many elderly persons this is something, this placement in a retirement home is a “bête noire”. Because it means total decline for them.” (P25/PrimCP).*

The other codes within this category were less salient but taken together they pointed to the fact that sufferings were emerging here from a kind of fear of going on living in a state of unbearable decay. Not only was there a fear of potential future suffering but this anticipation itself was a source of current suffering that would lead to a wish to die.

### Life is over

This category was mentioned by more than one-third of the participants. It was often described as being tired of life. Although it was often linked to other sufferings, it needed to be differentiated because it really referred to a fully aware reasoning: “My life has come to an end”. Here an example:

*“He tells me: “Do you understand? My world shrinks, you can do whatever you want. ( …*) *I know that I will be soon coming to an end. I hope everything is going to be alright and that someone will help me if not.” This is an objective observation. He is 91 years old.” (P20/EV).*

It is in this category that we found what we can call a kind of “pure” existential suffering. A suffering without identification of any other related ones. Only four of twenty-six participants talked about it and it was described in four out of sixty-three stories. An *EXIT* volunteer illustrated it:

*“But it concerns more the people in the retirement homes. Except this woman who clearly said that she didn’t want to find a spouse again and that her life was finished. That she had a beautiful life, with her husband, that she had raised her children, that she had grandchildren who were going well. So she had done it all and said “so I can leave now””. (P19/EV*).

It sounds like this woman had accomplished what she had to and was ready to die. No physical decline, no dependency. A little bit of loneliness maybe.

### Loss of social significance

Loss of social significance was mentioned by eight out of twenty-six participants and was especially described as a feeling of uselessness. Here is an illustration of how this feeling could be experienced as a deep suffering:

*“I also think that there is something else we didn’t talk about: this feeling of uselessness. Everything that refers to « me feeling useful », or « me feeling useless », « not serving purpose », is a big suffering.” (P5/PallCP).*

The loss of usefulness in family and society was described by participants as linked to the process of aging. And, as mentioned before for the code “Hurt self”, many participants pointed out the fact that society, our culture, might be partly responsible for these feelings such as expressed by a primary care provider:

*“( …*) *the big existential suffering for many people is this feeling of useless (ness). It doesn’t happen in the cultures where elderly remain integrated in the family, where they can even look after the grandchildren or participate in the activities. Those people (in Switzerland) are lonely.” (P25/PrimCP).*

### Loss of hope for a better future

Loss of hope for a better future being a cause of a life that is not worth living anymore had the same relevance as the loss of social significance. As one can imagine, the description of what a better future is, is highly personal and plural. This category refers to a feeling that the future will be worse than the present life. Here is an example of a conversation between an *EXIT* volunteer and someone he helped to die:

*“Well, I have to (request AS).” And I told her: “How is that? Why do you have to?” And she told me: “Yes I have to because I don’t have remission anymore.” Yes, it’s true. “Her diagnosis was clear, her remission was finished, her disease reappeared and she did not want to live through that.” (P19/EV).*

This woman wanted to requested AS because all she could expect from this point was suffering.

### Being a financial burden

The fact that being a financial burden could be a cause of existential suffering and lead to a AS request was stated by six out of twenty-six participants. It was often described as one more form of suffering on top of an already distressful daily life. Like other categories and codes, being a financial burden was described as closely related to the way society perceives old age, dependency or physical decline such as explained by an *EXIT* volunteer:

“*what those people generally fear is loneliness, and the fact of becoming a burden and being a high cost to society, as we can hear it and read it in newspapers. They also talk about this, and I always tell them “It’s not a reason. You worked; you don’t have to worry because you are expensive. You’re no more expensive than anyone else.”” (P18/EV).*

This participant highlights the fact that the concern of becoming a burden might be caused by media’s perception in these people.

### Loss of pleasurable activities

The loss of pleasurable activities was the least mentioned category and was often linked to other categories. However, it was important to highlight it because the loss of pleasures and joys of life was directly described as leading to a life that isn’t worth living anymore. Here is an illustration:

*“She found that it was not enough to be more or less fit again. And, above all, she gave up visiting Museums in Europe and she loved to do this in the past. So, she came back one day to terminate her life simply because her goals in life couldn’t be achieved any more. Because of physical or psychological problems.” (P12/PallCP)*.

In the story of this patient, life wasn’t worth living any longer mainly because her condition prevented her from enjoying museum visits, which used to be the greatest pleasure in her daily life. Hence, life did not make sense anymore for her.

## Discussion

One of the purposes of this study was to deepen the understanding of existential suffering from the perspectives of professionals (palliative and primary care providers) and Right-to-die organization volunteers when it is part of the request for AS, especially in a country like Switzerland, where it is legal. Our study found that existential suffering included physical decline and its consequences, loneliness, fear of the future, life is over, loss of social significance, loss of hope for a better future, being a financial burden and loss of pleasurable activities, all of which were intertwined with the impression that life had lost its meaning and/or was not worth living anymore.

Suffering coming from a “diminished perception of one’s own image” or from a “loss of self-identity” was always linked to physical decline, which could be a decline in the physical ability to invest life, a profound alteration in physical appearance, or both. In a society that gives great importance to physical appearance, as aging represents a slow loss of a culturally self-identity because one stops to be a productive and contributing member of society, physical decline leads to an even deeper fracture of identity. The loss of vitality separates one from a past life in which one could enjoy the body and depend on it. It also shuts the door on desires and hopes for the future.

According to our participants, society was somehow responsible at least in part for existential suffering. Perceptions of old age or physical decline are influenced by social contexts, they shape the way we see ourselves, and this can lead to deep suffering. A form of internalised ageism could be at work in these situations. [19] More pragmatically, lack of the sort of social support required to maintain relationships, a sense of self, and well-being may have played a role as well. [20]

Finally, we found that in most cases existential suffering consisted of different, and sometimes compounded, losses of the dimensions of life. Requests for suicide assistance in such cases were described as emanating from persons who were as if shut out from components of their own existence; a finding similar to the description by Sjöberg and colleagues of older patients’ narratives of being “disconnected from life”. [21] Despite this, a minority of cases did seem to represent what might be termed “pure” existential suffering, or a feeling that life was meaningless or over, without identification of any specific restrictions in the components of it. This was present in just four of sixty-three stories. These findings are relevant to the status of existential suffering as a possible justification for AS. Cases where life is -sometimes severely- restricted are morally significantly different from those where this is not the case. Existential suffering as a motive for suicide assistance requests often leads to controversial issues. This is partly due to questions regarding whether existential suffering is sufficiently severe, rather than a name given to situations where a decision to ask for death may have been made too lightly. Our findings suggest that most suicide assistance requests for reasons of existential suffering are made by people who are indeed suffering in readily recognizable ways. This is also what the few data from EXIT right-to-die organization on motives for AS requests tend to demonstrate. Even though existential suffering might be present in all requests, and this is something current data does not capture, a serious illness is always present. [22] Suicide assistance requests motivated by “pure” existential reasons raises different issues. Such cases are of course controversial, and viewed alternately as irrational, as cases where the true causes of suffering remain unrecognized, or as instances of “existential maturity” in facing our own mortality. [23] Our findings suggest that these situations do exist but only count for a small number of AS requests motivated by existential suffering. In these rare cases, the only justification, recognized by the Swiss law, for offering such assistance is to not have selfish motive and to respect the Federal Narcotics Act. The law actually does not specify requirements in terms of suffering. However, this year, the SAMS (Swiss Academy of Medical Sciences), which provides healthcare professionals with recommendations on ethics issues, published new guidelines on the management of dying and death. They stated that the assisting person has to verify five requirements, including that the “symptoms of disease and/or functional impairments are a source of intolerable suffering” for the patient. [24] For the first time, the institution provided healthcare professionals with more specific requirements in terms of suffering for requesting AS.

By probably providing the first in-depth published account on this studied group in this particular legal framework, our study contributes to the literature in several ways. It adds content to the definition of existential suffering. Existential suffering is not solely related to being tired of living. It comprises different forms of suffering, and sometimes combines them. As the recent integrated literature review concludes: “Although ambiguity in these definitions may be justifiable given the broad range of similarity in terminology, clinical clarity may be necessary given the current challenges of treating this form of suffering, and a general consensus that it has not been well defined or treated is also required”. [9] Clinical clarity is essential. It could provide a kind of “toolkit” to people concerned by this question. Based on our findings, we could for example imagine a questionnaire investigating at least these eight categories of existential suffering. This would help care providers and volunteers from “right-to-die organizations” to better understand the suffering experienced by the persons they are taking care of and, in this way, help them to propose more personalized and more appropriate care. Recognizing existential suffering to be plural could help to identify the different actors needed to assist individual patients.

This study has several limitations. First, as in all qualitative research, some intrusion of the researcher’s biases is inevitable. To reduce this effect, some of the interviews were double-coded and all the codes and categories were discussed together. Furthermore, an on-going and reflective research journal recorded the first researcher’s impressions to make sure to stay as close possible to what the participants wanted to share. Our sample size was small, and our participants were recruited exclusively in the French-speaking part of Switzerland. Our results cannot be generalized. However, as our sample of participants is quite representative of the people concerned by the question, it would be interesting to see whether our findings are transferable to other palliative care units, to other primary care providers and to other “right-to-die organizations” in Switzerland. As such, it might help to build a consensus on evaluating and treating existential suffering. Finally, our study focused on the perspectives of care providers and *EXIT* volunteers, and not on those of people requesting AS. We found, however, that their perspectives were not so distant from the ones of people actually requesting AS, particularly regarding the aspects of loneliness, dependency, hopelessness, loss of life’s meaning and loss of self-identity. [5, 6, 25]

## Conclusions

Representations by the palliative care and primary care providers and volunteers from *EXIT* on existential suffering are multiple. They include the notion of physical decline and its consequences, loneliness, fear of the future, life is over, loss of social significance, loss of hope for a better future, being a financial burden and loss of pleasurable activities. To our knowledge, no study had been conducted yet on the perspectives of these people in a country like Switzerland, where AS is legal and where different groups of people have to confront it and try to respond to it. Our study might be helpful in providing a better understanding of existential suffering to and help identify a wider range of alternatives to offer these suffering people, rather than simply thinking of limiting the conditions for an acceptable AS.

## Additional file

Additional file 1: **Table 1.** *Interview guide.* The semi-structured interview guide used to collect data. **Table 2.** *Participant characteristics* Number of participants and their demographics. **Table 3.** *Results.* Representations of existential suffering by the palliative care and primary care providers and volunteers from *EXIT* right-to-die organization. (DOCX 17 kb)

## Abbreviations

AS: assisted suicide
*CCER*: Commission Cantonale d’Ethique de la Recherche (Swiss Cantonal Ethics Committee)
EV: *EXIT* Volunteer
*EXIT*: name of a swiss right-to-die organization
PallCP: Palliative Care Providers
Pn: Participant number
PrimCP: Primary Care Providers
SAMS: Swiss Academy of Medical Sciences
