# Supplementary material for: A qualitative study on existential suffering and assisted suicide in Switzerland
Source: BMC Med Ethics. 2019 May 14;20:34. doi: 10.1186/s12910-019-0367-9 (PMC6515594; doi:10.1186/s12910-019-0367-9)
Supplement: Supplementary file 1 — Table 1. Interview guide. The semi-structured interview guide used to collect data. Table 2. Participant characteristics Number of participants and their demographics. Table 3. Results. Representations of existential suffering by the palliative care and primary care providers and volunteers from EXIT right-to-die organization. (DOCX 17 kb) [file 12910_2019_367_MOESM1_ESM.docx]

**ADDITIONAL FILES**

**Table 1- Interview guide**

| ***TOPIC / THEME*** | ***MAIN QUESTIONS*** | ***FOLLOW-UP QUESTIONS*** |
| --- | --- | --- |
| **Experience with existential suffering and assisted suicide** | Did you already receive requests for assisted suicide having existential suffering as a reason ?   - If yes, can you tell me more about it ? - In your opinion, why were the reasons of this request “existential” ? - What finally happened to this person ? | When you have been confronted to this existential suffering, did you explore it ?   - If yes, how ? - If no, why ? |
| **Difficulties** | Do you feel any difficulties when you face these kinds of requests ?   - If yes, which ? | Do you think you have the tools to confront them ?   - If yes, which ? - If no, what would you need ? |
| **Opinion** | Do you think that existential suffering is a justifiable reason for requesting assisted suicide ?   - Why ? |  |
| **Propositions** | 1. What should we propose to people requesting assisted suicide with a reason of existential suffering ?  - Which alternatives ?  1. What would be your role ?  - Who else do you imagine having to take care of existential suffering when it’s part of the request for assisted suicide ? | 1. Do you think it is your role to explore existential suffering ?  - If no, whose role is it ?  1. If the person doesn’t change his/her mind, how would you ensure that there is nothing else to do ? |

**Table 2 – Participant characteristics**

*N total = 26 participants*

*n* = number of participants*

|  | ***PALLIATIVE CARE PROVIDERS (n*)*** | ***VOLUNTEERS FROM EXIT (n*)*** | ***PRIMARY CARE PROVIDERS (n*)*** |
| --- | --- | --- | --- |
| ***People contacted*** | 17 | 6 | 4 |
| ***Participants*** | 16 | 6 | 4 |
| ***Female*** | 10 | 3 | 4 |
| ***Male*** | 6 | 3 | / |
| ***Age range*** | 43-62 | 49-80 | 52-63 |
| ***Years of experience in their domain (range)*** | 4-36 | 6-14 | 6-40 |

**Table 3 – Results**

*N total = 26 participants*

*N*= number of participants who* mentioned the category

*n* = number of participants who* mentioned the code

| ***CATEGORIES*** | ***CODES*** | ***n**** |
| --- | --- | --- |
| ***Physical decline and its consequences***  *N* = 21* | **Physical decline** | **15** |
|  | - Physical decline | 15 |
|  | - Physical pain | 2 |
|  | **Dependency** | **14** |
|  | - The refusal of dependency | 8 |
|  | - Physical dependence | 6 |
|  | - Loss of autonomy | 6 |
|  | **Hurt self** | **9** |
|  | - Diminished perception of one’s own image | 6 |
|  | - Loss of self-identity | 3 |
|  | - Loss of dignity | 2 |
| ***Loneliness***  *N* = 16* | Loneliness | 14 |
|  | Loss of loved ones | 4 |
| ***Fear of the future***  *N* = 15* | Fear of a terrible agony | 6 |
|  | Fear of being placed into a retirement home | 6 |
|  | Fear of the unknown | 3 |
|  | Fear of the hospital | 3 |
|  | Anxiety | 3 |
| ***Life is over***  *N* = 10* | Life is over | 10 |
| ***Loss of social significance***  *N* = 8* | Feeling useless | 8 |
|  | Loss of role | 1 |
| ***Loss of hope for a better future***  *N*= 8* | Loss of hope for a better future | 8 |
| ***Being a financial burden***  *N*= 6* | Being a financial burden | 6 |
| ***Loss of pleasurable activities***  *N*= 5* | Loss of pleasurable activities | 5 |
